# Transcriptomic and photosynthetic responses to grafting of the *Nod1* gene in nodulated and non-nodulated soybeans

**DOI:** 10.1093/g3journal/jkab209

**Published:** 2021-06-26

**Authors:** Qingyuan He, Shihua Xiang, Wubin Wang, Yingjie Shu, Zhengpeng Li, Songhua Wang, Lei Chen, Xiaoyan Yang, Tuanjie Zhao

**Affiliations:** 1 College of Life and Health Science, Anhui Science and Technology University, Fengyang 233100, China; 2 Soybean Research Institute/National Center for Soybean Improvement, Ministry of Agriculture/Key Laboratory of Biology and Genetic Improvement of Soybean/Ministry of Agriculture/National Key Laboratory of Crop Genetics and Germplasm Enhancement, Nanjing Agricultural University, Nanjing 210095, China; 3 Zigong Institute of Agricultural Sciences, Zigong 643000, China

**Keywords:** nodulation gene, soybean, grafting, photosynthesis, transcriptional expression

## Abstract

Legume plants form symbiotic relationships with rhizobia to convert N_2_ into ammonia, and the nodulation status can affect plant development including photosynthesis. However, the relationship between nitrogen fixation and photosynthesis during carbon and nitrogen metabolism remains unclear. This study was undertaken to unravel regulation of nodulation and photosynthesis using a spontaneous nonnodulated soybean mutant by grafting. The results of inheritance and gene mapping showed that the nonnodulated mutant was controlled by a recessive gene overlapped with the reported *rj1* locus, and might be a new *rj1* allele with 1 bp deletion in the fourth exon in comparison to the sequence of normal nodulation plants. According to grafting results, soybean nodulation is obviously determined by the roots, not the seedlings. Moreover, nitrogen content along with related metabolic enzyme activity, and photosynthetic capacity were enhanced by nonnodulated scions grafted with nodulated roots. Contrary results were obtained for nodulated scions grafted with nonnodulated roots. A total of 853 differentially expressed genes (DEGs) in the leaves and 1874 in the roots were identified by transcriptome analyses of the grafting treatments. We identified 285 differential gene ontology (GO) terms and 57 differential pathway terms identified in the leaves, while 856 differential GO terms and 207 differential pathway terms in the roots. Twenty DEGs interacting at translation level were selected, and the results of transcriptome analyses were verified by q-PCR. These findings indicated that the nodulation-related *Nod* allelic gene increases the nitrogen content of nonnodulated plants, which affects the enzymes involved in nitrogen metabolism, leading to changes in hormone levels and further regulation of photosynthesis and carbon metabolism.

## Introduction

Leaf nitrogen content is strongly related to photosynthetic capacity and other photosynthetic traits, including carboxylation capacity and electron transport rate (Kouki 2004). More than 90% of crop biomass is derived from photosynthetic products. Higher rates of photosynthesis in plants may be triggered by greater nitrogen allocation to ribulose bisphosphate carboxylase oxygenase (Rubisco) (Aerts and Chapin 2000). However, nitrogen is an element that limits plant growth in many ecosystems (Hermans *et al.* 2006). Plants often preferentially allocate their biomass to their root system at the expense of shoot growth when nitrogen is limited (Makino 2011). However, the photosynthesis of legumes can be promoted by symbiotic nitrogen fixation.

Leguminous plants can make their nitrogenous nutrients by forming symbioses with rhizobia. Nitrogen-fixing root nodules are formed between the legumes and rhizobia. *Rhizobium* cells enter a legume host where they gain carbohydrates in exchange for nitrogen (Gresshoff 2003; Oldroyd *et al.* 2011). The nodules provide an ideal microenvironment for the reduction of molecular nitrogen to ammonia by rhizobia and nutrient exchange between the symbionts. The formation of this symbiosis and the resulting nitrogen fixation are the result of chemical communication between the plant and the rhizobia (Jin *et al.* 2016; Ling *et al.* 2016). Nodulation is the basis of nitrogen fixation. Furthermore, complex networks of nodules exist between plants and rhizobia involving different cell layers and molecular signaling functions (Madsen *et al.* 2010).

Soybean is an important leguminous crop, providing 69 and 30% of plant protein and vegetable oil, respectively (Lam *et al.* 2010). Their nodulation traits are controlled by genetic loci, namely *Rj*(s) or *rj*(s), and inoculation with compatible *Bradyrhizobium* or *Ensifer* species in soybean (Hayashi *et al.* 2012). The *Rj*/*rj* genes are classified into three categories: (i) recessive alleles (*rj1*, *rj5*, and *rj6*) leading to nonnodulation phenotypes (Williams and Lynch 1954; Pracht *et al.* 1993), (ii) recessive alleles (*rj7*/nts1, nitrate-tolerant symbiosis 1) resulting in supernodulation phenotypes (Akao and Kouchi 1992; Harper and Nickell 1995), and (iii) dominant alleles of *Rj2, Rj3, Rj4*, and *Rfg1*, which inhibit nodulation in particular strains of *Bradyrhizobium* *japonicum USDA122*, *Ensifer* *elkanii USDA33*, *B. elkanii USDA61*, and *Ensifer* *fredii USDA257*, respectively (Hayashi *et al.* 2012). The *Rj/rj1* gene, having a sequence of 3.4 kb, is related to the lipo-oligo chitin LysM-type receptor kinase gene, and is located on soybean chromosome 2 (Indrasumunar *et al.* 2011). Gene *Rj1* expression controls the nodule number in soybeans, and overexpression of *GmNFR1*α can alleviate nodulation deficiency in acidic soil.

The homologous genes of *GmNFR1α* can also affect nodulation, however, the effects of these genes are not entirely consistent with the phenotype of nodulation (Zhang *et al.* 2007). Nodulation change may be related to alternative splicing of these genes (Williams and Lynch 1954). The regulation and expression mechanisms of these genes need to be further elucidated. Nodulation changes lead to nitrogen nutritional differences. It is not known whether these changes affect photosynthesis, carbon assimilation, and plant development, which warrants further study (Lawn and Brun 1974). The reciprocal grafting technique has been used to study the roles of shoots and roots in the uptake and transport of nutrients and nodulation in soybeans (Fujita *et al.* 1991; Perigio *et al.* 1993). A previous grafting study showed that differences in nodulation and N_2_ fixation are mainly controlled by root genotypes (Malik 1983) and that the supernodulating phenotype is controlled by the shoots (Perigio *et al.* 1993). Besides, the function of isoflavonoids in the process of nodulation has also been studied by grafting experiments (Cho and Harper 1991). The objectives of the instant research are to dissect regulation of photosynthesis and nitrogen metabolism during nitrogen fixation by grafting and transcriptome analysis using *Nod1* mutant.

## Materials and methods

### Plant materials and grafting experiment

*NN1138-2* (normal nodulation) and *T3791* (natural nonnodulated mutant) were used as the female and male parents, respectively. Their F_2_ generation (184 plants) was used as the mapping population. In the summer of 2015, the F_2_ plants were grown in pots (filled with field soil) to V4 stage (Fehr *et al.* 1971) and then transplanted into the field. Nodulation was investigated individually before transplanting. After self-pollination, F_3_ families were obtained and used for determining the genotype of F_2_ individuals.

Seeds of *NN1138-2* and *T3791* were surface-sterilized with 70% ethanol for 1 minute and 0.1% HgCl_2_ for 6 minutes, and then washed five times with sterile water. These seeds were planted in pots filled in sterile soil under greenhouse condition (natural light). Grafting was conducted at V1 stage (Fehr *et al.* 1971) with four treatments: *NN1138-2* roots + *NN1138-2* scion (NN), *NN1138-2* roots + *T3791* scion (NT), *T3791* roots + *NN1138-2* scion (TN), and *T3791* roots + *T3791* scion (TT).

*B.* *japonicum* strain USDA110 was grown at 28°C in a darkroom in a liquid yeast extract mannitol broth medium (YMB; pH 6.8) with moderate shaking (120 rpm). After 6 days, cells of USDA110 were amassed by centrifugation (4000 rpm, for 10 minutes), washed three times with sterile water, and diluted in water to an optical density of OD_600_ = 0.8. Each pot of grafted plants was inoculated with 50 ml of the bacterial suspension. The plants were watered and cultured with sterile water in a greenhouse with natural light until flowering time.

### DNA isolation and linkage mapping

Genomic DNA was extracted from young fresh leaves using the improved CTAB method (Saghai-Maroof *et al.* 1984). According to the simple sequence repeat (SSR) information from Soybase (https://soybase.org), 98 pairs of polymorphic primers were selected for genotypic screening in the parents and the F_2_ population. These SSRs were distributed across the whole genetic map. The PCR system, program, and production detection were the same as the description of He *et al.* (2015). Linkage map and location of nodulation gene were constructed and mapped using the inclusive composite interval mapping (ICIM) method (RSTEP-LRT-ADD model) in IciMapping V4.1 software (Wang *et al.* 2016).

### BSA analysis by genome sequencing

The nodulation and nonnodulation bulks were constructed with 30 homozygous F_3_ plants. DNA samples were treated using sonication method to generate 350-bp fragments. These DNA fragments were end-repaired, A-tailed, ligated with full-length adapter, and amplified by PCR. The PCR products were purified using the AMPure XP systemand the size distribution of the libraries was analyzed with an Agilent 2100 Bioanalyzer. Quantitative real-time PCR (qPCR) was used for accurate quantification. The libraries were sequenced on the Illumina HiSeq platform (Illumina, USA) at Genepioneer Biotechnologies (Nanjing, China). Raw sequence reads were filtered and the retained clean reads were aligned to the reference genome of Williams82 (Wm82.a2.v1). SNPs and InDels were detected using GATK software (McKenna *et al.* 2010).

### Candidate gene analysis

To identify the sequence differences in the candidate genes, total DNA was isolated from leaves of *NN1138-2* and *T3791* using the Plant DNA Kit (TianGen Biotech, Beijing, China), and the genomic segments of the *Glyma.02g270800* gene were sequenced. The primers used were designed by Primer Premier 5.0 (Supplementary Table S1). The target gene was amplified in sections, and then the products were sent to Personalbi (Shanghai, China) for sequencing, splicing, and assembly. The sequences of *NN1138-2* and *T3791* were aligned. The function of the mutated protein was predicted.

### Chlorophyll content estimation, photosynthesis, and nitrogen metabolism

Chlorophyll content was determined by spectrophotometry. Net photosynthetic rate (P_N_), transpiration rate (Tr), intercellular CO_2_ concentration (Ci), and stomatal conductance (Gs) were measured using the portable photosynthesis system LI-6400XT in the first flowering period between 10.00 and 11.00 a.m. Monoamine oxidase (MAO) and nitrate reductase (NR) activities were assayed according to aldehyde phenyl hydrazone colorimetry (Leagene Biotech) and an *in vitro* method, respectively. Dried samples were triturated to powder and their N content was determined by the Kjedahl method. Nitrate nitrogen (NO_3_-N) and ammoniacal nitrogen (${\text{NH}}_{4}^{+}$-N) were determined by the salicylic acid and indophenol blue spectrophotometry methods, respectively.

### RNA-sequence and transcriptome analysis of grafting

A total of 12 samples from the four grafting treatments were collected and RNA-sequence analysis was conducted on the leaves, primitive roots of the rootstock, and the new scion roots. Total RNA was isolated from tissues using Trizol reagent (Invitrogen Life Technologies, Ambion^®^, UK). Transcriptomic libraries were constructed using NEBNext RNA super-speediness library preparation kits, including mRNA isolation and fragmentation, first-strand cDNA synthesis, second-strand cDNA synthesis, cDNA purification, end-repair, and dA-tail addition, adaptor ligation, segment size selection (300–400 bp), library enrichment, and purification. The quality assessment and quantification of the libraries was performed. Then, sequencing was carried out on Illumina HiSeq platform (Beijing Ori-gene Science and technology, LTD.).

The sequencing results were aligned against the *Williams 82* genome sequence (https://phytozome.jgi.doe.gov/pz/portal.html) using tophat-2.0.10. The percentages of saturation and coverage were analyzed using RSeQC (Wang *et al.* 2012). Novel genes were forecasted using Cufflinks and annotated by comparison with the Swiss-prot database. The abundance of transcripts, or gene expression, was calculated using FPKM (as follows). Correlations between treatments were measured in terms of the level of gene expression. Differences in gene expression among different samples were identified using the criterion of Trapnell *et al.* (2013). Alternative splicing of genes was analyzed by the rMATS method (Shen *et al.* 2014). gene ontology (GO)/KEGG enrichment analysis of differentially expressed genes was conducted based on a hypergeometric test, taking *P *<* *0.05 as the threshold of significance (Young *et al.* 2010).

$$\text{FRKM}=\frac{\text{Unique}\_\text{map}\;\text{ped}\text{fragment}’s\;\text{number}\text{of}a\text{transcript}\times 10^{9}}{\text{Total}\text{Unique}\_\text{map}\;\text{ped}\text{fragment}’s\;\text{number}\times \text{base}\text{number}\text{of}a\text{transcript}\;}$$

Q-PCR was performed to validate the RNA-Seq results of 20 differentially expressed genes (DEGs) with interaction at translation level of RNA-Seq analysis differed across the 12 samples. Primers for q-PCR were designed using Primer Premier 5.0 (http://frodo.wi.nit.edu/primers) (Supplementary Table S1). Expression levels of these genes were normalized according to the tubulin gene (NCBI accession No. AY907703). Gene expression levels were quantified using the relative quantification method (ΔΔCT).

## Results

### Genetic study for the nodulation trait and determination of the allele associated with nodulation gene

#### Genetic study for the nodulation trait

Table 1 shows the results of segregation of nodulation/nonnodulation in the offspring and their parents. The *NN 1138-2* and *T3791* parents exhibited nodulation and nonnodulation traits, respectively. All F_1_ plants of *NN 1138-2 *×* T3791* exhibited nodulation, indicating that nodulation is controlled by the dominant gene/allele. Segregation of nodulation to nonnodulation in the F_2_ population fitted a 3:1 ratio. The F_3_ population segregated at a ratio of 1 nonnodulation: 2 segregation: 1 nodulation (thus, 1:2:1). Therefore, the nonnodulation/nodulation trait was controlled by one mendelian factor.

**Table 1:** Segregation of nodulation in the NN 1138-2 × T3791 crossing

Generation	Total plants	Nodulation	Nonnodulation	Segregation	*χ* ^2^	Expected ratio	*P*
NN1138-2 (P_1_)	100	100	0	0	—	—	—
T3791 (P_2_)	100	0	100	0	—	—	—
F_1_ (P_1_ × P_2_)	90	90	0	0	—	—	—
F_2_	1272	971	301	0	1.212	3:1	0.271
F_3_	847	222	205	420	0.740	1:1:2	0.691

#### Conventional QTL analysis using the F_2_ and F_3_ populations

**Figure 1:**
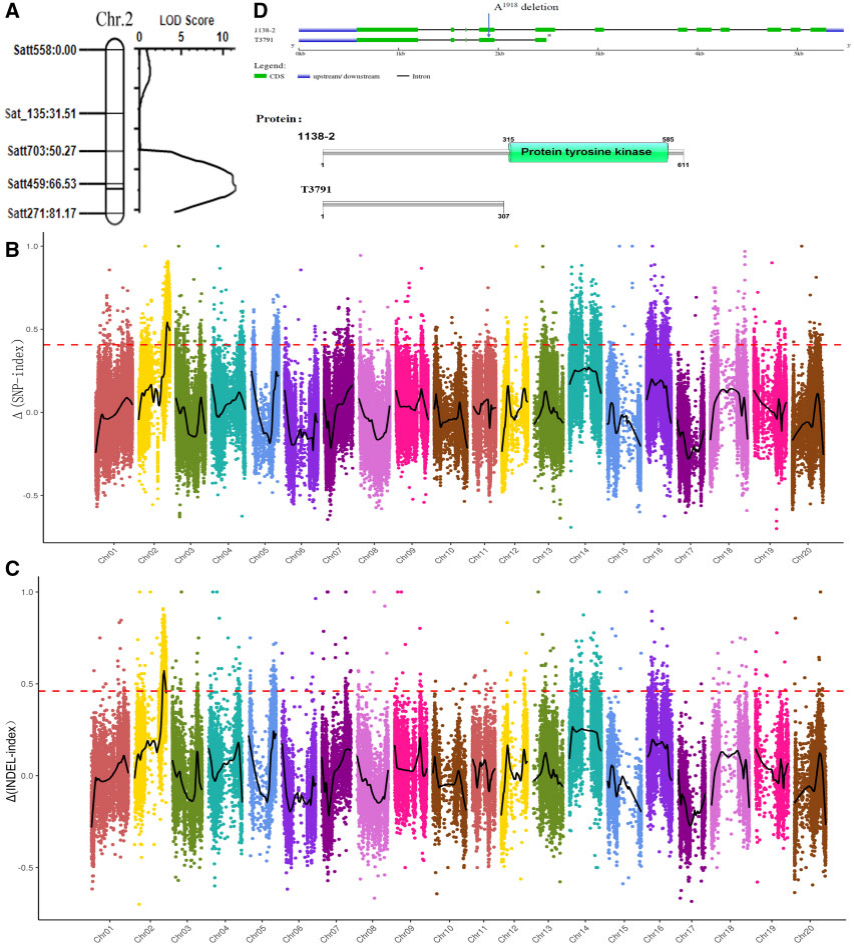
Location of the putative Nod1 gene using SSR and BSA sequence. (A) Initial location of the putative *Nod1* gene. (B) Δ(SNP-index) association analysis of the *Nod-1* candidate position in whole genome. (C) Δ(INDEL-index) association analysis of the *Nod-1* candidate position in whole genome. (D) The mutation of glyma.02g270800 gene resulted in the termination of gene translation. The rings from outside to inside represent the genome, SNP density distribution, INDEL density distribution, and SV (INS, DEL, and INV) density distribution, respectively. The *x*-axis represents the position of 20 chromosomes, the *y*-axis represents the Δ(SNP-index) or Δ(INDEL-index), the scatter points represent the value of that Δ(SNP-index) or Δ(INDEL-index), black curves represent lines of best-fit, the pink dotted lines represent the 99% threshold line.

A genetic map was constructed using 98 SSR markers from the 20 linkage groups in the 184 F_2_ individuals. This map spanned 1480.31 cM. The proportion of nodulated/nonnodulated F_3_ plants was considered as the phenotype of the F_2_ individuals. The putative allele controlling nodulation (*Nod1*), located between Satt459 and Satt271 on Chr.02, was identified by the ICIM method in IciMapping software (Figure 1A).

#### Detection of QTL using BSA-seq analysis

Four libraries (one for nodulation, one for nonnodulation, and two for parents) were constructed and subjected to whole-genome resequencing using Illumina HiSeq 2500. A total of 173,266,284, 111,850,845, 214,170,082, and 208,865,083 clean reads were obtained from the *NN1138-2*, *T3791*, nodulation, and nonnodulation bulks, respectively. The quality of all sequencing data ≥Q30 was >88.39%. The sequencing reads of the four samples were aligned to the genome of Williams82. The mapping rate of the resulting four sequences was >98.33% and the quality values of the reads mapped to the reference genome were >Q50. The average genome coverage depth ranged from 36.48 to 69.27 (Table 2). A total of 435,977 SNPs and 117,878 Indels were identified as differential between the nodulation and nonnodulation pools, including homozygous, and heterozygous SNPs. Some 326,922 SNPs and 67,002 Indels were obtained after filtration according to the following criteria: (i) the genotypes of the mixed pools of offspring were inconsistent, and (ii) the sequencing depth was not less than 5× in the two pools. Circos was used to analyze the distribution and plot it against the genome positions of the polymorphisms. This indicates that the distribution of the SNPs and Indels across the chromosomes was not uniform in the two parents and two pools (Supplementary Figure S1, A and B).

**Table 2:** Sequencing statistics and genome coverage

Samples	Clean reads	Clean data		Mapped reads				Coverage	
		Base	%≥Q30	Number	%	Duplication (%)	Quality	Depth	Ratio
NN1138-2	346532568	51979885200	90.75	341335168	98.50	27.66	57.97	55.74	99.00
T3791	223701690	33555253500	90.29	219473204	98.19	23.11	58.12	36.48	99.26
Nodulation bulk	428340164	64251024600	89.84	424786338	99.17	30.18	58.01	69.27	99.34
Nonnodulation bulk	417730166	62659524900	88.39	413948386	99.09	30.82	58.00	68.34	99.34

The SNP-index and INDEL-index represent the frequency of parental alleles in the population of bulked individuals. The Δ(SNP-index) and Δ(INDEL-index) values were calculated to determine associations between significant genomic positions. Peak regions above the threshold value (99%) were considered as regions where nodulation association may be located. The candidate nodule gene (*Nod1*) was located between 42358660 and 48572118 on Chr.02 using SNP and between 43030619 and 48324669 on Chr.02 using INDEL analysis (Figure 1, B and C). These are consistent with the findings of SSR, which indicates that the localization result is reliable.

### Gene annotation within the candidate region

There were 682 predictive genes in the candidate region. Of these, 661 genes were annotated by GO, KEGG, and Swissprot (Supplementary Table S2). These genes were identified by GO enrichment as belonging to three main categories: biological processes, molecular function, and cellular components (Supplementary Table S3). Pathway analysis linked the genes to different metabolic pathways (Supplementary Table S4) including the *Glyma.02g270800* (*Nod1*) gene (KO04626). The *Glyma.02g270800* gene has 12 exons and is part of the transmembrane receptor protein serine/threonine kinase signaling pathway (GO: 0007178). The Glyma.02g270800 sequences were compared by sequencing analysis. There was a difference of one base pair (A) deletion at the position of 1918 bp in the fourth exon between the two parents, which leads to frame-shifts with premature termination of only 307 amino acids in *T3791*, while the normal protein has 611 amino acids in parent *NN1138-2* (Figure 1D).

### Effects on photosynthesis and nitrogen metabolism by mutual grafting

**Figure 2:**
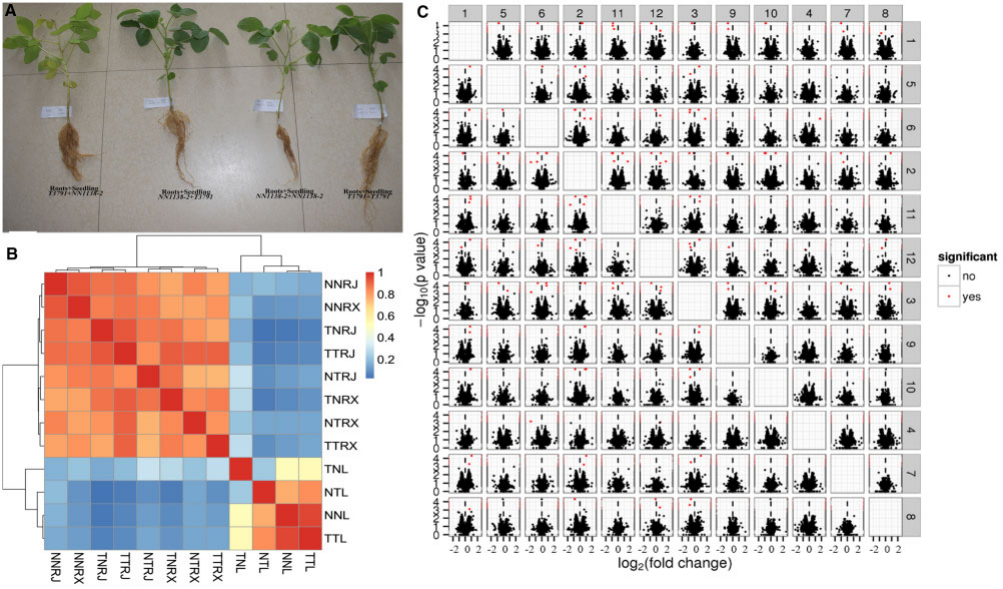
Phenotypes, gene expression difference, and correlation of the different grafting (A) Phenotypes. (B) Gene expression difference and correlation. (C) Volcano plot. NNL (1), NTL (2), TTL (3), TNL (4), NNRX (5), NNRJ (6), TNRX (7), TNRJ (8), TTRX (9), TTRJ (10), NTRX (11), and NTRJ (12) represent for leaves of NN, leaves of NT, leaves of TT, and leaves of TN, new roots of NN, primitive roots of NN, new roots of TN, primitive roots of TN, new roots of TT, primitive roots of TT, new roots of NT, primitive roots of NT, respectively. Each point represents a gene, the *x*-axis represents the $\;{\text{log}}_{2}^{\left(\text{fold}\;\text{change}\right)}$ in two samples, the *y*-axis represents the –log_10_P^-value of gene expression^. Red dots represent significantly.

After grafting, the nodulation characteristic was unchanged. The primitive roots (roots of rootstocks) of *NN1138-2* still showed nodulation, while the primitive roots of *T3791* showed no nodulation. The new roots emerging from scions of the *NN1138-2* roots showed nodulation, while *T3791* showed no nodulation. There were significant differences in leaf color among the four treatments. The leaf color of the NT treatment was the greenest, followed by the two self-grafting treatments, while the leaf color of the TN treatment was more yellow (Figure 2A).

The chlorophyll content, net photosynthetic rate (P_N_), transpiration rate (Tr), stomatal conductance (Gs) of NT treatment were significantly creased, while intercellular CO_2_ concentration (Ci) exhibited significantly decreased. On the contrary, the chlorophyll content, and P_N_, Tr, and Gs were considerably reduced in TN treatment, while Ci was increased. There was no significant difference between the two self-grafting treatments (Table 3). These results showed that nodulation can promote photosynthetic capacity and that nonnodulation led to a decrease in photosynthetic capacity.

**Table 3:** Photosynthetic rate and chlorophyll content of the four treatments

Treatment	*P*_n_ (µmol m^−2^s^−1^)	*T*_r_ (mmol m^−2^ s^−1^)	*C*_i_ (µL L^−1^)	*G*_s_ (mol m^−2^ s^−1^)	Chl *a* (mg g^−1^)	Chl *b* (mg g^−1^)	Chl (mg g^−1^)
NN	20.23 ± 2.47 b	4.63 ± 0.06 bc	251.33 ± 31.66 bc	381.00 ± 63.66 b	1.20 ± 0.13 b	0.53 ± 0.06	1.73 ± 0.09 b
NT	31.83 ± 0.55 a	7.10 ± 0.85 a	280.33 ± 7.51 b	616.67 ± 50.34 a	2.05 ± 0.13 a	0.71 ± 0.24	2.76 ± 0.36 a
TN	13.17 ± 1.62 c	5.73 ± 0.49 b	326.00 ± 13.75 a	563.33 ± 61.42 a	0.35 ± 0.10 d	0.64 ± 0.04	0.99 ± 0.07 c
TT	17.10 ± 2.89 b	4.07 ± 0.67 c	226.00 ± 9.17 c	236.67 ± 47.71 c	0.96 ± 0.13 c	0.67 ± 0.06	1.63 ± 0.08 b

*P*_n_, net photosynthetic rate; *T*_r_, transpiration rate; *C*_i_, intercellular CO_2_ concentration; *G*_s_, stomatal conductance; Chl *a*, Chl *b*, and Chl, content of chlorophyll *a*, chlorophyll *b*, and total chlorophyll, respectively. Data are the mean value of three replicates ± standard derivation (SD). Values with different letters indicate significant difference among treatments (*P* < 0.05).

The results showed that the distribution and metabolism of nitrogen were different in different tissues. There were significant differences between the roots and the aboveground parts in all five traits (the content of the total nitrogen, ${\text{NH}}_{4}^{+}$-N, and NO_3_-N, the activity of MAO and NR). There were also significant differences between the primitive roots and the new roots in terms of ${\text{NH}}_{4}^{+}$-N, NO_3_-N, and NR Compared with TN treatment, the NT treatment significantly augmented the total nitrogen, ${\text{NH}}_{4}^{+}$-N, and NO_3_-N contents in the roots and aboveground parts. Similar results were obtained for MAO and NR activity. The results showed that there were no significant differences in the MAO activity in new roots or the NR activity in leaves (Table 4). These results presented that the nodulated root of *NN1138-2* increased the nitrogen content by nodulation and nitrogen fixation, while *T3791* had its own regulation mechanism to ensure nitrogen balance and promote the formation of chlorophyll to improve its photosynthesis capacity under conditions of low nitrogen supply due to its lack of nodulation, such as by reducing NR activity.

**Table 4:** Nitrogen distribution and metabolism in the different tissues of the grafting treatments

Trait	Sample type	Treatments				Average
		NN	NT	TN	TT	
Total nitrogen concentration (%)	Aboveground part	2.75 ± 0.17 ab	2.88 ± 0.21 a	2.56 ± 0.21 b	2.68 ± 0.21 ab	2.72 ± 0.21 a
	Primitive roots	1.33 ± 0.07 ab	1.40 ± 0.05 a	0.99 ± 0.16 c	1.29 ± 0.14 ab	1.25 ± 0.19 b
	New roots	1.47 ± 0.19 a	1.47 ± 0.16 a	1.05 ± 0.16 b	1.38 ± 0.33 ab	1.34 ± 0.26 b
${\text{NH}}_{4}^{+}$ -N concentration (mg g^−1^)	Aboveground part	20.43 ± 2.44 b	34.54 ± 2.56 a	14.56 ± 2.31 b	19.93 ± 1.53 b	22.37 ± 7.96 c
	Primitive roots	78.83 ± 7.30 b	87.84 ± 3.02 a	51.20 ± 2.06 c	50.54 ± 5.07 c	67.10 ± 17.76 a
	New roots	32.27 ± 2.69 b	43.66 ± 4.18 a	34.55 ± 2.55 b	41.00 ± 4.29 a	37.87 ± 9.95 b
NO_3_-N concentration (mg g^−1^)	Aboveground part	0.13 ± 0.03 b	0.23 ± 0.04 a	0.04 ± 0.01 c	0.12 ± 0.03 b	0.13 ± 0.07 c
	Primitive roots	0.49 ± 0.07 b	0.66 ± 0.11 a	0.26 ± 0.05 c	0.40 ± 0.06 b	0.45 ± 0.17 a
	New roots	0.33 ± 0.05 b	0.42 ± 0.08 a	0.16 ± 0.05 c	0.32 ± 0.07 b	0.31 ± 0.11 b
Monoamine oxidase activity (NO_2_ µg g^−1^ h^−1^)	Leaves	3.06 ± 0.37 ab	3.41 ± 0.46 a	2.58 ± 0.39 b	3.00 ± 0.46 ab	3.01 ± 0.47 a
	Primitive roots	2.37 ± 0.30 ab	2.63 ± 0.34 a	2.02 ± 0.35 b	2.31 ± 0.20 ab	2.33 ± 0.35 b
	New roots	2.31 ± 0.28 a	2.31 ± 0.31 a	1.91 ± 0.40 a	2.27 ± 0.26 a	2.20 ± 0.32 b
Nitrate reductase activity (µg g^−1^ h^−1^)	Leaves	3.12 ± 0.39 a	4.62 ± 0.46 a	1.76 ± 0.21 a	3.19 ± 0.41 a	3.17 ± 1.11 c
	Primitive roots	37.61 ± 3.48 b	74.58 ± 13.70 a	16.92 ± 0.99 c	34.68 ± 6.27 b	40.95 ± 22.88 a
	New roots	12.37 ± 0.50 b	30.70 ± 4.58 a	9.85 ± 1.24 b	12.74 ± 1.72 b	16.42 ± 8.96 b

Data are the mean value of three replicates ± standard derivation (SD). The first four columns are the compare among four treatments. The last column compares average of different sample types. Values with different letters indicate significant difference among treatments (*P* < 0.05)

### Analysis of transcriptome expression for grafting

RNA-Seq data from the Illumina HiSeq platform produced 39.77–54.91 million clean reads with ≥60 bp and quality values ≥ Q30 and 5.55–7.64 G clean bases for 12 samples. There were 25.60–47.83 million reads that passed the filtering criteria and mapped uniquely to the reference genome of Williams82.a2 (Supplementary Table S5). The gene expression was basically saturated and the distribution of reads was relatively uniform in the genome.

Raw digital gene expression counts were normalized using a variation of the fragments/Kb/million (FPKM) method. Analysis of gene expression in the different sample groups showed that the number of expressed genes ranged from 31,937 to 37,004 (Supplementary Table S6). Analysis of the relationship between the four samples showed less difference between the two self-grafted samples, followed by those of NT and TN. The results indicated that the roots significantly affected the gene expression of the leaves. The aboveground parts of the plant can also affect the gene expressions of the roots. Therefore, the new root gene expression level of grafted scions of *T3791* was less affected by the primitive root system, while that of *NN1138-2* was more affected by the primitive root system (Figure 2B).

Comparative analysis of the different treatment libraries revealed significant expression changes in 0 to 1608 genes. Among them, significant DEGs in the leaves ranged from 70 to 270, and those in the roots ranged from 0 to 241 (Supplementary Table S6). A volcano plot of gene expression differences between the samples is shown in Figure 2C. The greater the absolute value on the *x*-axis, the more significant difference in fold-expression between the two samples. The greater the value on the *y*-axis, the more significant difference of expression is.

### Genetic association mapping of DEGs between the grafting

There were five DEGs in the leaf grafting treatments within the genetic mapping association region, and six in the roots. The DEGs of the roots were *Glyma02g245600* (gibberellin-regulated family protein), *Glyma02g248000* [calcium-dependent lipid-binding (CaLB domain) family protein], *Glyma02g256800* (cytochrome P450 superfamily protein), *Glyma02g270800* (chitin elicitor receptor kinase), *Glyma02g293700* (unknown), and *Glyma02g304500* (ADP glucose pyrophosphorylase). The DEGs of the leaves were *Glyma02g244600* (MYB domain protein/regulation of transcription by RNA polymerase II), *Glyma02g261200* (temperature sensing protein-related), *Glyma02g293700* (unknown), *Glyma02g299500* (integral component of membrane), and Glyma02g307500 (unknown). The *Glyma02g270800* gene was not expressed in the leaves in any of the four treatments. The highest expression level of *Glyma02g270800* was detected in the new roots of the NN and TN treatments. The second-highest level of expression of *Glyma02g270800* was detected in the primitive roots of the NT treatment. The expression levels in the other treatments were very low.

### GO and KEGG pathway enrichment analyses of DEGs

**Figure 3:**
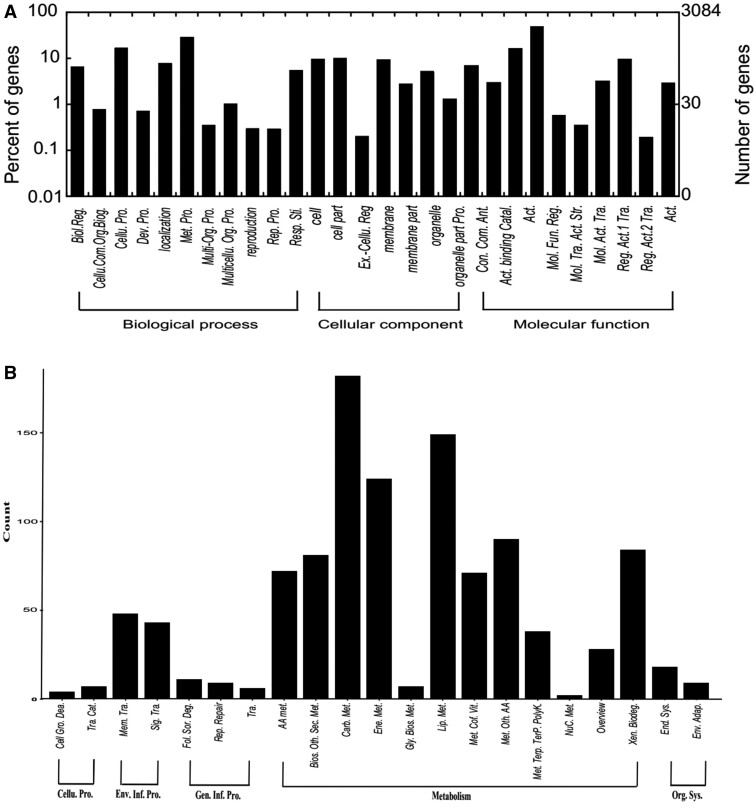
Results of GO classification and Pathway analysis (A) GO classification, (B) Pathway. The *x*-axis represents GO classification or Pathway, the *y*-axis represents the number of significantly different terms. Biol. Reg., Biological regulation; Cellu. Com. Org. Biog., Cellular component organization or biogenesis; Cellu. Pro., Cellular process; Dev. Pro, Developmental process; Met. Pro., Metabolic process; Multi-Org. Pro., multi-organism process; Multicellu. Org. Pro., multicellular organismal process; Rep. Pro., reproductive process; Resp. Sti., response to stimulus, Ex.-Cellu. Reg, extracellular region; Pro. Con. Com., Protein-containing complex; Ant. Act., antioxidant activity; Catal. Act., Catalytic activity; Mol. Fun. Reg., Molecular function regulator; Mol. Tra. Act., Molecular transducer activity; Str. Mol. Act., structural molecular activity; Tra. Reg. Act., transcription regulator activity; Tra. Reg. Act., translation regulator activity; Tra. Act., transporter activity; Cellu. Pro., Cellular processes; Cell Gro. Dea., Cell growth and death; Tra. Cat., Transport and catabolism; Env. Inf. Pro., Environmental information processing; Sig. Tra., Signal transduction; Mem. Tra., Membrane transport; Gen. Inf. Pro., Genetic information Processing; Tra., Translation; Rep. Repair, Replication and repair; Fol. Sor. Deg., Folding, sorting and degradation; NuC. Met., nucleotide metabolism; Xen. Biodeg., Xenobiotics biodegradation; AA met., Amino acid metabolism; Bios. Oth. Sec. Met., Biosynthesis of other secondary metabolites; Carb. Met., Carbohydrate metabolism; Ene. Met., Energy metabolism; Gly. Bios. Met., Glycan biosynthesis and metabolism; Lip. Met., Lipid metabolism; Met. Cof. Vit., Metabolism of cofactors and vitamins; Met. Oth. AA, Metabolism of other amino acids; Met. Terp. TerP. PolyK., Metabolism of terpenoids and polyketides; Org. Sys., Organismal Systems; End. Sys.: Endocrine system; Env. Adap., Environmental adaptation.

The DEGs caused by grafting were categorized into 3 categories: cellular components, molecular functions, and biological processes (Figure 3A and Supplementary Table S7). Among these, 3487 GO terms were identified with significant differences, ranging from 0 to 111 per sample. A total of 285 significant terms were identified among the leaf samples, ranging from 27 to 68 per sample. Among these 124, 51, and 110 GO terms were categorized into biological processes, cellular components, and molecular functions, respectively. There were 778 significant terms were identified among the roots, ranging from 0 to 58 per sample, and among these, 191, 186, and 401 terms were categorized into biological processes, cellular components, and molecular functions, respectively (Supplementary Table S8). The significant GO terms were categorized into 28 functional groups, including biological processes (11), cellular components (8), and molecular functions (9) (Figure 3A). For biological processes, the following were recognized: biological regulation, cellular component organization or biogenesis, cellular processes, developmental processes, localization, and metabolic process.

Significantly enriched metabolic pathways and signal transduction pathways were identified by enrichment analysis of the DEGs (Figure 3B and Supplementary Table S9). A total of 1083 pathway terms with significant differences were identified, ranging from 0 to 44 per sample. Among these, 56 significant terms were identified in the leaves, ranging from 4 to 21, While 139 were identified among in the roots, ranging from 0 to 13 (Supplementary Table S10). Further, the five main pathway categories were identified, including metabolic pathways (928), environmental information processing (91), organismal systems (37), genetic information processing (26), and cell processes (11). The metabolic pathways included 12 metabolic types, such as carbohydrate metabolism (182), lipid metabolism (149), energy metabolism (124), metabolism of other amino acids (90), and xenobiotic biodegradation, and metabolism (84), and so on. A total of 56 significant terms were identified among the leaf samples, ranging from 0 to 14, including metabolism (51), environmental information processing (4), and genetic information processing (1). In roots, 139 significant terms were identified, ranging from 0 to 10, including cellular processes (7), environmental information processing (22), genetic information processing (11), metabolism (91), and organismal systems (8) (Supplementary Table S9). The differential grafting treatments could affect the expression of genes related to receipt of environmental information, affect nitrogen metabolism through the nodulation response, and lead to changes in carbon metabolism.

### Network analysis of DEGs in grafting treatments

The crucial factors affecting the metabolism of nodulation and nitrogen fixation were investigated by transcriptome analysis of the grafted plants. These included amino acid metabolism (16 out of 56 in leaves and 10 out of 139 in roots), flavonoid biosynthesis (1 in leaves and 9 in roots), carbohydrate metabolism (11 in leaves and 11 in roots), plant hormone signal transduction (4 in leaves and 5 in roots), and nitrogen metabolism (2 in roots), etc. (Supplementary Table S9). The coordination of these metabolic pathways leads to differences in nitrogen metabolism and photosynthesis.

There are often interactions between the different RNAs and proteins occurring in organisms. Based on the differential genes, we analyzed and revealed the interactions between differentially expressed transcripts and proteins from different perspectives. The correlation between the genes was screened using threshold correlation coefficient values of >0.99 or <−0.99 and a significance value of *P *>* *0.05. A total of 9637 pairs of transcripts and 975 pairs of proteins were interacted with each other based on the analysis of gene expression in all 12 samples (Supplementary Tables S11 and S12). The Chr.20 had the lowest interacting transcripts, having only 25, with the highest on Chr.08 having 104 interacting transcripts. The Chr.12 had the lowest interacting proteins having only 2, with the highest on Chr.10 having 14 interacting proteins (Supplementary Tables S11 and S12). Gene transcripts and protein up- or down-regulation were compared based on comparisons between all groups. Network diagrams of the co-expressed transcripts and proteins involved in different gene interactions were plotted using Cytoscape (Supplementary Figure S3, A and B).

**Figure 4:**
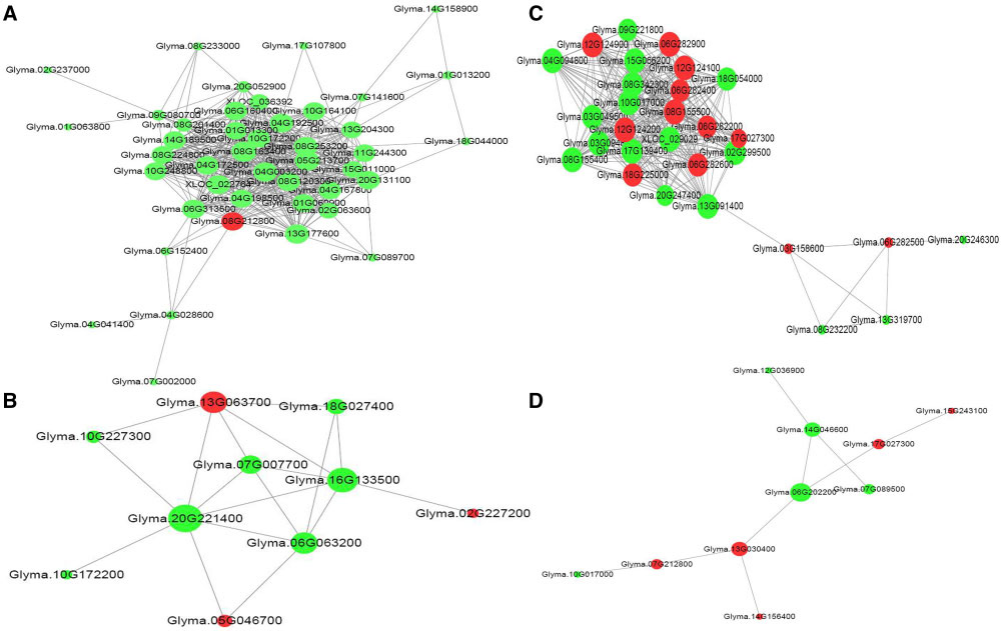
Network diagram of the different gene co-expression and proteins (A) Co-expression network in roots. (B) Proteins network in roots. (C) Co-expression network in leaves. (D) Proteins network in leaves. Red nodes represent up-regulation, green nodes represent down-regulation. The size of the node represents the number of gene co-expressions. The edges represent the co-expression relationship of the gene interaction, solid lines represent the positive correlation between genes, and dotted lines represent the negative correlation between the genes.

A total of 434 pairs of transcripts and 18 pairs of proteins interacted with each other according to the analysis of gene expression level in the eight root samples (Supplementary Tables S13 and S14). Network diagrams of the co-expressed transcripts and proteins involved in the different root gene interactions are presented in Figure 4, A and B. A total of 276 pairs of transcripts and ten pairs of proteins interacted with each other according to the analysis of the gene expression level in the four-leaf samples (Supplementary Tables S15 and S16). Network diagrams of the co-expressed transcripts and proteins involved in the different leaf gene interactions are listed in Figure 4, C and D.

Ten proteins were interacting with DEGs in the roots: Glyma.02g227200 (fatty acid desaturase), *Glyma.05g046700* (arginosuccinate synthase family), *Glyma.06g063200* (casein lytic proteinase B4), *Glyma.07g007700* (ATPase E1-E2 type family protein/haloacid dehalogenase-like hydrolase family protein), *Glyma.10g172200* (UDP-glycosyltransferase superfamily protein), *Glyma.10g227300* (multidrug resistance-associated protein 14), Glyma.13g063700 (ATP binding cassette subfamily B19), *Glyma.16g133500* (P-loop containing nucleoside triphosphate hydrolases superfamily protein), *Glyma.18g027400* (cyclophilin-like peptidyl-prolyl cis-trans isomerase family protein), and *Glyma.20g221400* (ammonium transporter). *Glyma.20g221400* is the gene responsible for ammonia transport; its differential expression affects amino acid metabolism and transport, and ATP energy transport (Figure 4B).

Ten proteins were interacting with DEGs in the leaves: *Glyma.06g202200* (heat shock protein), *Glyma.07g089500* (Ribosomal protein L12/ATP-dependent Clp protease adaptor protein ClpS family protein), *Glyma.07g212800* (nitrite reductase), *Glyma.10g017000* (glycosyl hydrolase), *Glyma.12g036900* [NAD(P)-binding Rossmann-fold superfamily protein], *Glyma.13g030400* (aldehyde dehydrogenase), *Glyma.14g046600* (Lycopene beta/epsilon cyclase protein), *Glyma.14g156400* (alcohol dehydrogenase), *Glyma.15g243100* (solanesyl diphosphate synthase), and *Glyma.17g027300* [NAD(P)-binding Rossmann-fold superfamily protein]. *Glyma.14g046600* is a chlorophyll-related gene and it regulates the expression of oxidoreductase and energy metabolism-related enzyme genes (Figure 4D).

### Validation of RNA-Seq data by qRT-RCR

To validate the results of the expression patterns among the grafting treatments by RNA-Seq, we used q-PCR to analyze the expression levels of 20 DEGs with the interaction between at translation level. Although the log_2_-fold values of RNA-Seq showed slight differences to those of the q-PCR analyses, the expression levels detected by the two methods were basically the same (Supplementary Figure S4). The results showed that the interaction of DEGs was verified using q-PCR.

## Discussion

### Nodulation gene mapping

In this study, the segregation of nodulation genes conformed to the Mendelian law of a single dominance gene. The nodulation gene was located on the same position in Chr.02 in both the F_2_ and F_3_ populations based on the results of two methods: SSR markers and high-throughput whole-genome re-sequencing. The SNP-index, and Indel-index also obtained the same results via the BSA-Seq method. Hence, the mapping results are quite reliable. There are 682 candidate genes in this region, including the *Nod1* gene (Indrasumunar *et al.* 2011). Based on previous studies and our sequencing results, there is 1-bp deletion in nonnodulated parent *T3791* compared to nodulated parent *NN1138-2* (Supplementary Figure S2) and terminated protein translation*.* Hence, the variation in nodulation could be caused by this variation in sequence.

### Effects of the *Nod1* gene on nodulation, photosynthesis, and nitrogen metabolism in grafted treatments

Previous studies have shown that the grafting of soybean scions and roots can affect plant nitrogen metabolism, hypernodulation regulation (Hamaguchi *et al.* 1992), and cadmium accumulation (Megumi *et al.* 2007). Control of the supernodulating phenotype resides in the shoots, while the nonnodulating phenotype is regulated by roots (Perigio *et al.* 1993). The factors causing the changes in the scions and roots could also affect the isoflavonoid content to regulate nodulation (Cho and Harper, 1991). The study also showed that nodulation is entirely controlled by roots and not affected by the scions, and that the nodulation is only controlled by the root genotype. The current results suggested that the *Nod1* gene is solely expressed in roots. Nodulation can be controlled by certain genes using short- and long-distance signals to achieve equilibrium between cell proliferation and differentiation. The nodule primordials are regulated by shoot-root signaling known as autoregulation of nodulation (AON) (Suzaki and Nishida 2019). *GmNARK* expression in the leaf has a major role in long-distance communication between nodules and lateral root primordials (Searle *et al.* 2003). The results indicated that the *Nod1* gene is only expressed in roots, so it is considered that the *Nod1* gene is a short-distance signal gene.

The differences in nodulation in underground root systems can also affect photosynthesis and nitrogen content. The grafted nonnodule seedlings with nodule roots had the highest photosynthetic capacity and nitrogen content. Conversely, grafted nodule seedlings with nonnodulated roots had the lowest photosynthetic capacity and nitrogen content. Similar results were also obtained for NH_4_-N, NO_3_-N, MAO, and NR. These results suggested that the nodulation gene *Nod1* affects nitrogen metabolism and subsequently photosynthesis. The nitrogen fixation ability of nodules significantly increased the content of nitrogen, and enhanced the MAO and NR activity in the process of nitrogen metabolism. The high content of nitrogen may promote the increase of chlorophyll content and improve photosynthesis capacity.

### Effect of *Nod1* gene expression in grafted plants

RNA-Seq technology provides a powerful way to determine gene functions, regulatory networks, and expression profiles. The technology has been widely used to study the global expression profiling and regulation of various traits in soybean, such as nodulation (He *et al.* 2018), bacterial leaf pustulation (Kim *et al.* 2011), glabrousness (Hunt *et al.* 2011), and lipid biosynthesis (Chen *et al.* 2012). Nodulation occurs by the interaction of a series of genes and can affect the expression of other genes. The LysR-family transcriptional regulatory protein triggers the horizontal gene transfer (HGT) process in response to plant flavonoids (Ling *et al.* 2016). This study identified a total of 853 DEGs among leaves and 1874 among roots, 285 differential GO terms among leaves and 856 among roots, and 57 differential pathway terms among leaves and 207 among roots in the grafting treatments. Nodulation genes affect a series of genes by altering nitrogen metabolism, such as those related to photosynthesis, plant hormone signal transduction, and so on. The *Nod1* gene (*Glyma.02g270800*) was not expressed in leaves, on the contrary, it was highly expressed in the new roots of *NN1138-2*. This indicates that *Nod1* gene may be possible for nodulation; hence, further study will be needed to elucidate mechanism underlying this gene and the phenotype. A regulatory network was formed based on the ten DEGs in leaves, including the regulation of photosynthesis, energy metabolism, and so on. Another regulatory network was developed based on the ten DEGs in roots, including the regulation of energy metabolism, ammonium transportation, etc., due to differences in nodulation. These networks give a clue about possible interaction in regulating nodulation in soybean. Therefore, functional validation of few genes (especially hub-genes and highly interconnected genes in the network) are recommended for future study.

### Signaling regulation of the *Nod1* gene

Previous studies have identified several host legume genes involved in Nod factor (NF) perception and subsequent symbiotic signal transduction, bacterial infection, nodule organogenesis, and the regulation of nitrogen fixation (Radutoiu *et al.* 2003; Kouchi *et al.* 2010; Suzaki and Nishida 2019). The *Nod1* gene is a LysM-type receptor kinase gene with putative Nod factor receptor components in soybean (Indrasumunar *et al.* 2011). Nodulation and nitrogen fixation consume energy from plant photosynthesis and form a feedback autoregulation system to balance nitrogen fixation and photosynthesis (Heckmann *et al.* 2006; Reid *et al.* 2011). This study showed that the *Nod1* gene affects nodulation, thereby affecting nitrogen metabolism and ammonium transportation, leading to changes in photosynthesis in the different treatments. The nodulation/nonnodulation root grafting treatments caused differences in nitrogen levels, resulting in changes in NR activity, thus affecting the expression of hormone-related genes. Co-enzymatic gene expression in the energy metabolism pathway was altered through network regulation, and changing the rate of photosynthesis in leaves. The results provided here offer deeper understanding of the regulation of nodulation in soybean, which will aid in our future breeding efforts to breed for prolific nodulation genotype.

## Conclusions

The results in this study suggest that the *Nod1* gene located Chr.02:43030619-48324669 bp region. There was one base pair (A) deletion at the position of 1918 bp in the fourth exon which leads to frame-shifts with premature termination. The gene increased nitrogen content and photosynthetic capacity when nonnodulated scions were grafted onto nodulated roots; in contrast, nitrogen content and photosynthetic capacity decreased when nodulated scions were grafted onto nonnodulated roots. 853 DEGs were identified among leaves and 1874 among roots, and there were 285 and 856 differential GO terms among leaves and roots, respectively. Also, through KEGG pathway enrichment analysis, 57 and 207 differential pathways were detected in roots and leaves, respectively. As a long-distance regulatory nodulation gene, *Nod1* increases nitrogen content after nodulation, which affects enzymes related to nitrogen metabolism, leading to changes in hormone levels and further regulation of photosynthesis and carbon metabolism.
